# Efficacy of Multiple Exercise Therapy after Coronary Artery Bypass Graft: A Systematic Review of Randomized Control Trials

**DOI:** 10.31083/j.rcm2405141

**Published:** 2023-05-09

**Authors:** Md. Moneruzzaman, Wei-Zhen Sun, Geoffrey J. Changwe, Yong‑Hui Wang

**Affiliations:** ^1^Rehabilitation Center, Qilu Hospital of Shandong University, 250012 Jinan, Shandong, China; ^2^Department of Cardiac Surgery, Qilu Hospital of Shandong University, 250012 Jinan, Shandong, China; ^3^Department of Cardiovascular and Thoracic Surgery, National Heart Hospital, 10101 Lusaka, Zambia

**Keywords:** cardiac rehabilitation, coronary artery disease, CABG, exercise therapy, post-operative care

## Abstract

**Background:**

Coronary artery bypass graft (CABG) is intended to restore myocardial perfusion and alleviate morbidity among patients suffering from coronary artery disease. Due to procedural complexity, and anesthetic medications, post-operative complications are more prevalent, requiring the integration of rehabilitation strategies. This review aimed to determine the effect of single and multiple exercise therapy on rehabilitation after CABG surgery.

**Methods:**

We conducted a systematic search of databases (EBSCOhost, Scopus, PubMed, and Web of Science) from 01 January 2000 to 15 September 2022. The protocol of this systematic review is registered to PROSPERO.

**Results:**

We found nine randomized control trials composed of 599 CABG patients. In-patient cardiac rehabilitation (CR), a combination of inspiratory muscle training, mobilization, active upper and lower limb exercise, and aerobic exercise as multiple exercise therapy, found significant improvement in 6-minute walking distance (6MWD) than single exercise therapy (breathing exercise) at discharge and follow-up (moderate quality evidence). Contrary, multiple exercises group compared to single exercise groups did not improve the peak volume of oxygen (${\mathrm{VO}}_{2}$) at discharge. Still, significant improvement was found at follow-up (moderate quality of evidence). On the other hand, the out-patient CR made up of high-intensity inspiratory muscle training, upper and lower limbs resistance training, and aerobic exercise as multiple exercise therapy significantly improved 6MWD and peak ${\mathrm{VO}}_{2}$ at discharge (High-quality evidence).

**Conclusions:**

Our review revealed that multiple exercise therapy significantly improves functional and exercise capacity in in-patient and out-patient cardiac rehabilitation settings than single exercise therapy, but more than double exercise therapy protocol may be inefficient for improvement of quality of life. Inspiratory muscle training and resistance training in exercise therapy protocols significantly supplant the outcome, which requires further investigation.

## 1. Introduction

Coronary artery bypass graft (CABG) is a recommended revascularization method. It is aimed to sufficiently restore myocardial perfusion and alleviate morbidity amongst patients suffering from coronary artery disease (CAD) [1]. The prevalence of performed CABG in the USA is about 6% to 14% due to significant CAD; over 200,000 CABG operations are performed annually [2, 3, 4]. As for Asia, in mainland China mortality rate of CABG is 1.9%; the 2020 annual report estimated that 330 million people have CAD [5]. Despite its high effectiveness, CABG performed via midline sternotomy retains several adverse effects, such as deep sternal wound infection, chest discomfort, stroke due to procedural complexity, and anesthetic medications [1, 4]. The aforementioned adverse effects, combined with psychological deviations like persistent depression, post-operative cognitive dysfunction, and delirium, lead to hospital re-admission after a successful CABG. Literature reports that 10% of the cases are re-admitted within one-month post-procedure [6].

Cardiac rehabilitation (CR), as defined in various literature, aims to increase exercise tolerance, improve functional capacity, and reduce cardiac morbidities [7, 8]. Studies reported that CR reduces hospitalization time (hazard ratio, 0.66; 95% CI, 0.63–0.69) and the risk of death by 4.2% after CABG [9]. In addition, CR significantly improves exercise tolerance (35%), increases high-density lipoprotein (HDL) cholesterol (12%), optimizes pulmonary oxygen uptake [1], and subsequently enhances the quality of life (QoL) [10]. Today, CR is a comprehensive therapy for CABG patients [11, 12, 13, 14]. Because of various challenges and patients preferences, the therapies can be center-based or home-based [15, 16, 17]. In addition, CR further focuses on pre-and post-operative education, post-procedural adverse effects such as thoracic pain, breathing difficulties, decreased mobility, deviation of typical physiological systems, and psychological and mental health such as anxiety and depression [18, 19]. Despite the benefits mentioned above, active participation in CR programs after CABG remains low, thus 35 to 40% [9, 20, 21]. Some published systematic reviews reveal the potential benefit of applying various therapies such as inspiratory muscle training, resistance training, aerobic exercise, and breathing exercises [14, 22, 23, 24]. For example, evidence from an randomized control trial (RCT); reports that a treatment plan involving low-intensity resistance exercise with early mobilization improves cardiac patients’ exercise capacity and endurance [25]. However, one literature report that the addition of breathing exercise to CR protocol does not alter the efficiency [26], yet, on the contrary, combined training for CABG patients shows greater effectiveness on pulmonary function [27].

Moreover, numerous studies illustrate that single exercise therapy (aerobic exercise, inspiratory muscle training) significantly improves CABG patients’ exercise capacity, functional capacity, and QoL [22, 23]. Contrarily, significant improvements were also demonstrated after multiple exercise therapy (a combination of several single exercise therapy) [25]. Single and multiple exercise therapy can be performed on CABG (on-pump or off-pump) patients’ after mechanical ventilation being weaned off, usually from the second post-operative day (in-patient) and after discharge from the hospital at a rehabilitation center-based (out-patient) or home-based for one to several weeks [14]. Therefore, multiple exercise therapy programs consume more time and money, requiring extra specific facilities (instrumental or center-based) or care (supervision). These paradoxical issues would impact a rehabilitation program regarding patients’ safety, participation, or overall recovery. Hence, the necessity of practicing multiple exercise therapy after CABG needs to be explored. The significance of adding one or multiple exercises or combined exercise training to existing CR for CABG patients remains a daunting question.

However, this systematic review intended to find and compare the effect of multiple and single exercise therapy on only CABG patient rehabilitation on functional and exercise capacity and quality of life.

## 2. Materials and Methods

This review presents data following the preferred reporting items for systematic reviews and meta-analyses (PRISMA) statement 2020 [28] and synthesis without meta-analysis (SWiM) guidelines [29]. The PRISMA and SWiM checklist is available in the supplementary material (**Supplementary Methods 1,2**). Only RCTs were considered for inclusion if they were composed of patients who exclusively underwent or awaited CABG and enrolled in CR, regardless of age or sex. Data were excluded if patients underwent or awaited CABG simultaneously with other cardiac operations or complications (i.e., arrhythmias, myocardial infarction, neuromuscular disorders). The protocol of this review is registered and published online (PROSPERO, Reg. No. CRD42021259327).

### 2.1 Search Strategy

**Fig. 1.**
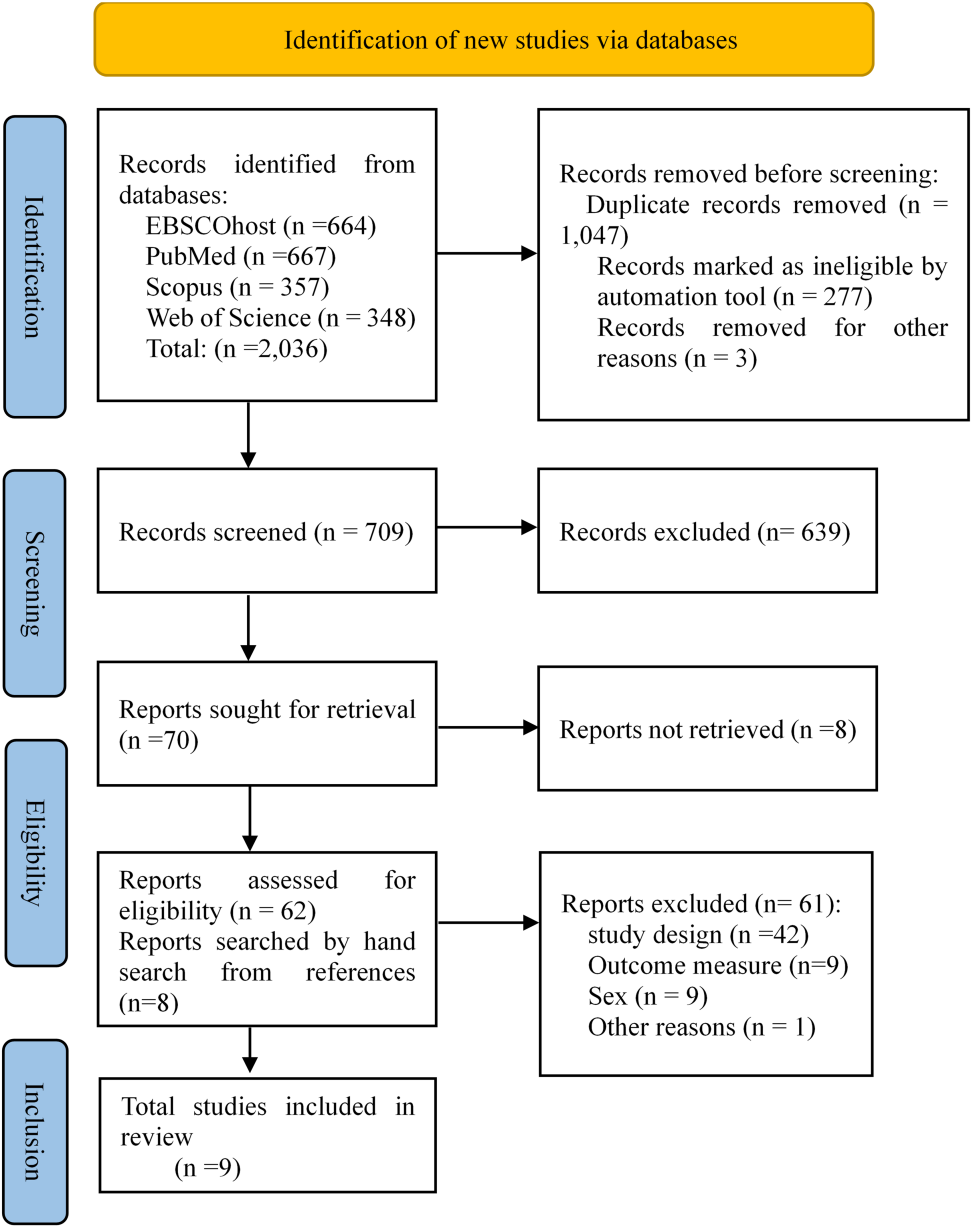
**PRISMA flow diagram for identification of studies from the databases**.

Using our institutional electronic database, we systematically searched academic journals from reputable search engines, including EBSCOhost, Scopus, PubMed, and Web of Science. The publication language was limited to English, Chinese, and Russian because the available authors were only fluent in those three languages. The search terms (e.g., “exercise”, “therap*”, “physical activity,” “rehab*”, “prehab*”, “post-rehab*”, “physical therapy,” “CABG”, “coronary artery bypass graft*,” “cardiac”) focused on published articles from 01 January 2000 to 15 September 2022 to assess the most recent studies. After an extensive screening of all eligible articles, only RCTs were considered (Fig. 1). A thorough search strategy is available (**Supplementary Table 1**). Additional studies were searched from the reference list through hand search for further reviewing and selection (**Supplementary Methods 3**).

### 2.2 Study Selection

Study selection and screening of article titles and abstracts were made using PICO(S) methods [30]. Considering PICOS, the selected CABG patients in CR reflected as a population, given exercise(s) therapy as intervention, in between exercise(s) therapy protocol(s) or without exercise(s) group as a comparison, while functional and exercise capacity, and QoL as an outcome(s) of a given RCT study design. A precise search results description is available (**Supplementary Table 1**). We merged the search results for all official databases using the reference management software ‘Zotero’ (version 6.0.20, Corporation for Digital Scholarship, Vienna, VA, USA) and discarded duplicates. Further, a citation management platform ‘Rayyan’ (https://rayyan.ai/) [31], was utilized to cross-check citations, inclusion, and exclusion. Author (MM) searched and extracted all the articles independently. Authors (MM) and (WS) checked and selected articles independently. Any conflict on data selection or exclusion was resolved by discussion with the author (GJC). Finally, the author (YW) made a final decision.

### 2.3 Data Collection and Synthesis

Omitted data from an already published study by Miozzo *et al*. [32]; Hirschhorn *et al*. [33]; and Busch *et al*. [34] was collected through direct contact and utilized (**Supplementary Methods 4,5,6**).

To effectively execute meta-analysis, we extracted numerical data from variables including functional capacity as 6-minute walking distance (6MWD), exercise capacity as peak volume of oxygen (${\mathrm{VO}}_{2}$), and QoL as short form 36 (SF-36) and other available questionnaires at discharge and follow-up. Further, baseline information such as age, sex, hypertension, diabetics, beta-blocker use, time of surgery, and length of intensive care unit stay was extracted and tabulated (**Supplementary Table 2**).

The synthesized data was reported as follows: continuous outcomes as mean difference (MD) and potential standard errors (SE) were converted to standard deviation (SD) using the Cochrane handbook. The studies were categorized and selected using the Cochrane handbook for a systematic review of intervention [35]. Studies were grouped according to the exercise intervention. We considered a core exercise protocol as a single exercise, such as aerobic exercise. If another core exercise, such as inspiratory muscle training used with a single exercise, we consider it a double exercise protocol; this way, we categorize all exercise protocols (**Supplementary Tables 3,4**). In this study, multiple exercises refer to more than one core exercise therapy. Exercise duration, intensity, time, and type differed among included studies with study settings. The meta-analysis result was not attainable due to the high heterogeneity assessed by the *$I^{2}$* value (>70%) and the lack of required data. However, we made minor changes to our protocol. We conducted a narrative synthesis with outcome data of all included RCT studies (**Supplementary Table 3**). Studies were analyzed for inter and intra-group effects using the Review Manager (RevMan) software (version 5.4.1, the Cochrane Collaboration, London, United Kingdom) and data presented in the forest plot. Where available, a significant *p*-value was considered for effect estimation.

### 2.4 Study Risk of Bias and Quality Assessment Tools

Level of certainty of evidence among studies evaluated by the GRADEpro guideline development tool (GDT) (https://gdt.gradepro.org). We assessed studies for quality using the ‘Physiotherapy evidence database (PEDro)’ scale [36] with a score scale of 10 points tabulated as follows: 9–10 as significantly high quality, 6–8 as good quality, 4–5 as fair, and <4 as poor quality. Further, the quality and risk of bias were assessed using the risk of bias 2 (RoB 2) tool (https://www.riskofbias.info) [37]. No studies were excluded based on quality. The tasks mentioned above were performed independently by (MM) & (WS) and cross-checked by (GJC) and (YW).

## 3. Results

### 3.1 Search Result

The initial search found a total number of 2036 relevant articles from the previously mentioned four search engines. The initial title and abstract screening isolated 62 articles, and full-text screening was reduced to 9 relevant articles [32, 33, 34, 38, 39, 40, 41, 42, 43]. The articles found through hand search were not included in the final analysis due to further inappropriateness with the protocol (**Supplementary Method 1**). We reported detailed findings of the included studies (Table 1, Ref. [32, 33, 34, 38, 39, 40, 41, 42, 43]).

**Table 1.** **Summary of all included articles**.

Author, Country, (n =)	Analyzed sample size, n; ${\mathrm{(Drop-out)}}^{a}$; Male/female	Self-rejection after meeting eligible criteria (N/Refused)	Age, mean (limits, years)	Treatment duration ${\mathrm{(Phase, P)}}^{b}$	Exercise frequency ${\mathrm{(Control group)}}^{c}$	Exercise Intensity	Exercise protocols	Outcomes
Han *et al*. 2022 [41], China (n = 140)	140 (16); 104/36	276/12	63.5 (≥50)	1 week (P1)	2x/Day	Borg RPE scale = 4/10, 10 repetitions, Arterial blood pressure <65 mmHg or >110 mmHg, heart rate <50 beats/minute or >120 beats/minute, respiratory rate <12 breaths/minute or >40 breaths/minute; pulse oximetry <88%	Health education, aerobic and breathing exercise, early mobilization	Activities of daily living, post-operative pulmonary complications
Eibel *et al*. 2022 [39], Brazil (n = 15)	15 (6), 12/3	43/9	62.3 (50 to 75)	1 week (P1)	2x/Day	15 to 20 breaths/min, 30% of MVC and MIP	Passive manual respiratory therapy, walking exercise, isometric hand grip resistance training, inspiratory muscle training	6-minute walk test, flow-mediated dilatation, Maximum inspiratory pressure, oxidative stress
Girgin *et al*. 2021 [40], Turkey (n = 50)	50 (0), 31/19	102/2	61.16	4 days (P1)	3x/Day	Modified Borg scale: 2–4, 1–2 METS exercises 10 repetitions	Lower-upper extremity exercises, Deep breathing exercises, Active Cycle of Breathing Techniques, Walking, Postural drainage	Lung function, Six-minute walk test, QoL
Zanini *et al*. 2019 [43], Brazil (n = 40)	40/39 ${\mathrm{(1)}}^{d}$; 29/11	382/76	58.5 (18 to 70)	≥6 days (P1)	2x/Day	20% of Maximum Inspiratory Pressure (1 to 4 cmH2O), Borg RPE scale = 11	Deep breathing, Active movement of ankle and wrist, Flexion of hip and knee, Plantar flexion in orthostatic posture, UL, and LL flexion up to 90°, 600 m walking from the stationary stage, stepping up and down	Functional capacity, respiratory muscle strength, lung function
						40 repetitions active mv of ankle and wrist, 10 breaths, walking stationary to 600 m; ( 100 m increase/day), 1 min to 30-sec rest in between exercises, UL and LL exe. 2 sets and 15 reps		
Miozzo *et al*. 2018 [32], Brazil (n = 24)	18 (6); 15/3	42/5	57.5 (30 to 70)	12 weeks (P2)	3x/Week	50%–80% of Maximum Inspiratory Pressure, 50%–80% of Peak HR, 10 to a maximum of 12 repetitions	40 min aerobic exercise, IMT, and AE in 3 phases, 50%–80% reserve Peak HR and maximum inspiratory pressure with 10 to 12 repetitions, each phase increase 10%, ergometric test, Peak HR and ${\mathrm{VO}}_{2}$ followed by Bruce protocol	Six-minute walk test, respiratory muscle strength, QoL
Santos *et al*. 2018 [38], Brazil (n = 24)	24 (0); 17/7	27/0	55.8 (45 to 65)	12 weeks (P2)	2x/Week	50%–80% maximal inspiratory pressure, Borg RPE scale = 11, 10–12 repetitions	30-minute walking on a motorized treadmill, Resistance exercises for upper limbs and lower limbs with dumbbells, shin guards, or elastic bands	functional capacity, lung function, respiratory muscle strength, QoL
Busch *et al*. 2012 [34], Germany (n = 173)	173 (23); 54/119	382/65	78.5 (75 to Older)	3 weeks (P1)	1x/Day (3x,2x/Week)	60% one Repetition Minimum, RPE scale 13; 8 to 12 repetitions 30 min/session	Walking, cycle ergometer, calisthenics, leg extension, leg press, leg curls using weight machines, and biceps curls using free weights	6-minute walk test, cardiopulmonary strength, Maximum isometric strength, Health-related QoL
Mendes *et al*. 2009 [42], Brazil (n = 74)	47 (27); 36/11	107/2	59 (not estimated)	5 Days (P1)	1x/ Day	2 to 4 Metabolic Equivalent of the test (MET), resting HR + 20 bpm, 10 to 15 repetitions, starts with 5 min and last days are 10 min	Deep breathing, coughing or huffing, Active-assistive exercises of the lower/upper extremities, ankles, and wrists bed inclined at 45°, in a sitting position at 90°, flexion-extension of the bilateral shoulder, elbow, wrist, knee, and ankle; adduction–the abduction of the hips, Orthostatic position, stairs climbing	Cardiac autonomous regulation
Hirschhorn *et al*. 2008 [33], Australia (n = 92)	92 (5); 80/12	117/3	62.9 (not estimated)	4 Weeks (P1)	2x/Day	Oxygen saturations >92%, Modified Borg RPE Scale on moderate intensity, walking repetitions, 20 repetitions of breath	Walking exercise normal and progressive, stair climbing, active movement, Health education, Postural drainage	6-minute walk test, vital capacity, Health-related QoL

^a^ Reason for drop-out: Miozzo *et al*. [32] 2018: Problem with work and transport, peripheral vascular disease; Busch *et al*. [34] 2012: General clinical conditions, excessive demand; Mendes *et al*. [42] 2009: Refuse to continue, poor quality of HR signal Death (n = 7); Hirschhorn *et al*. [33] 2008: did not attend follow up, hospitalized during a follow-up appointment. ^b^ P1, Inpatient cardiac rehabilitation; P2, Outdoor or rehabilitation center-based cardiac rehabilitation. ^c^ Study reported a control group with a different frequency than the intervention group. ^d^ After 30-day post-discharge, analyzed samples were 39. n, number; N, Total eligible patient; 6MWD, Six-minute walking distance; ${\mathrm{VO}}_{2}$, Volume of oxygen; RPE, Rating of perceived exertion; HR, Heart rate; bpm, beat per minute; QoL, Quality of Life; MVC, maximum voluntary contraction; MIP, Maximum inspiratory pressure; UL, Upper Limb; LL, Lower Limb; IMT, inspiratory muscle training; AE, Aerobic exercise.

### 3.2 Study Characteristics

We analyzed baseline data from 599 CABG patients (**Supplementary Table 2**); Seven articles [32, 33, 34, 38, 39, 40, 43] assessed functional capacity on 6MWD; Four studies [32, 34, 38, 43] assessed exercise capacity on peak ${\mathrm{VO}}_{2}$; one study [42] evaluated functional capacity on heart rate variability. Lung function was assessed in four articles [38, 40, 41, 43]. For the assessment of QoL, six studies applied various approaches, including (I) SF-36 [32, 33, 40], (II) Brazilian version SF-36 [32], (III) Portuguese version of Minnesota Living with Heart Failure Questionnaire (MLHFQ) [38], and (IV) MacNew questionnaire for health-related quality of life (HRQL) [34].

### 3.3 Exercise Intervention 

Of the analyzed studies, seven [33, 34, 39, 40, 41, 42, 43] conducted an in-patient CR with a minimum of 5 days and a maximum of 4 weeks of treatment duration. Two studies [32, 38] were out-patient CR; both studies had a 12 weeks treatment duration. Meanwhile, only two articles [33, 43] reported follow-up results. The average exercise frequency was one or 2-times/day, performed 3 to 5 or 7 days/week, and breathing exercise was performed at a frequency of 10–20 breaths. Each exercise was composed of approximately ten repetitions in 2–4 sets. The exercise intensity was measured using maximum inspiratory pressure (20% and 50%–80%) with oxygen saturation above 92%; peak heart rate at 80%, while perceived exertion using the Borg scale at a moderate level, treatment protocols followed CR guidelines [44] (Table 1). **Supplementary Table 3** tabulated all exercise groups and available outcome data from included article. We have calculated the odd ratio to estimate between-group effects. Our results showed that multiple exercise therapy contains inspiratory muscle training (IMT) and resistance training changes the odds of exercise effect in a group compared to aerobic exercise therapy. Therefore, we found that the minimum significant exercise duration was 5-days with an intensity of 20% of maximum inspiratory pressure, performed once per day at a frequency of 8 to 10 repetitions.

### 3.4 Results of Outcomes

#### 3.4.1 Functional and Exercise Capacity

**Fig. 2.**
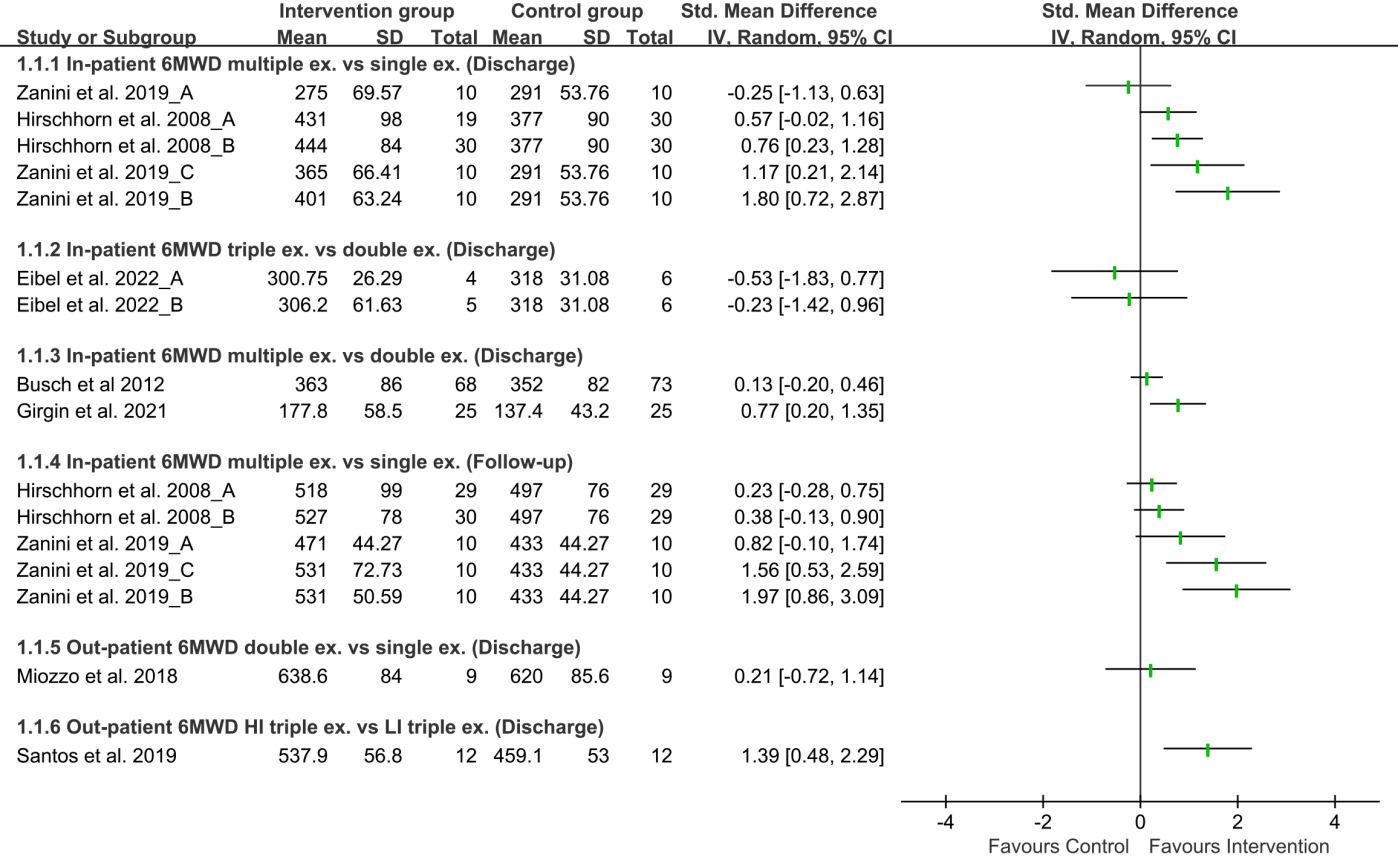
**Forest plot of functional capacity on 6-MWD**. This figure compares combined exercises with intervention group and control group for 6MWD. Standardized mean difference of each study indicated effect of exercise (6MWD, six-minute walking distance; ex, exercise; vs., Versus; SD, Standard deviation; IV, Inverse variance; CI, Confidence interval; HI, High-intensity; LI, Low-intensity).

**Fig. 3.**
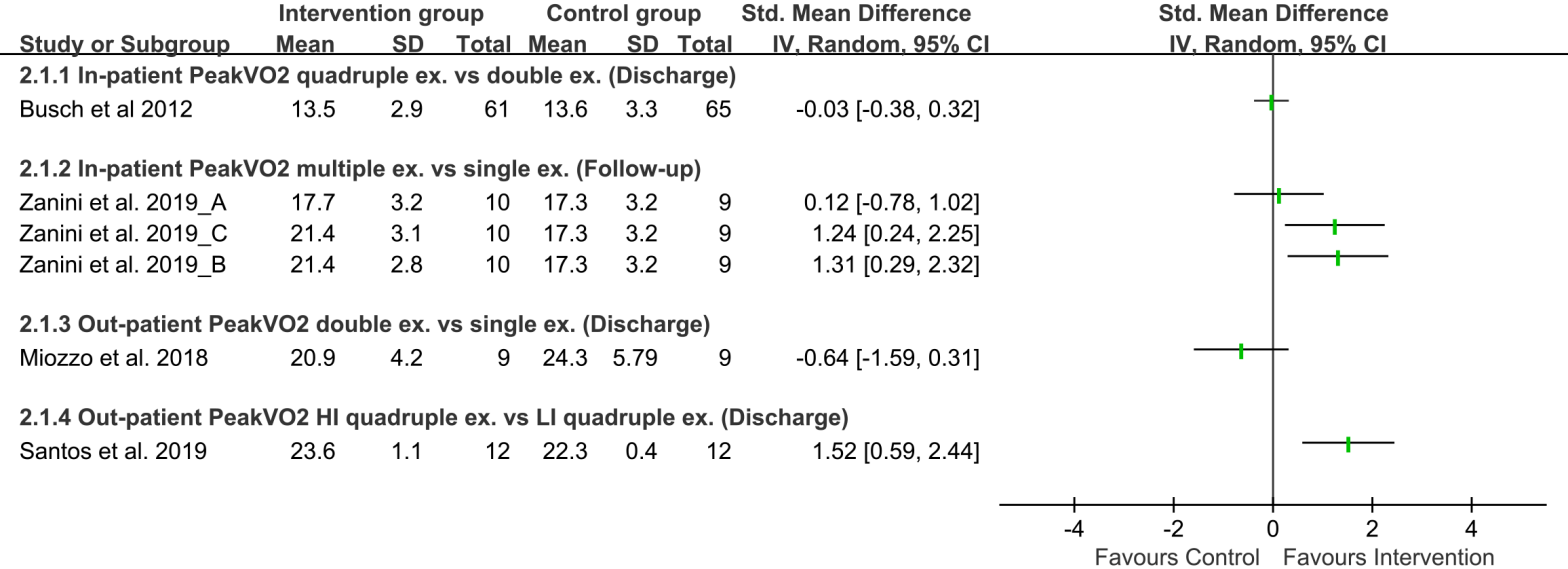
**Forest plot of exercise capacity on Peak ${\mathrm{VO}}_{2}$**. This forest plot shows the comparison between combined exercises with intervention group and control group for peak ${\mathrm{VO}}_{2}$. Standardized mean difference of each study indicated effect of exercise (${\mathrm{VO}}_{2}$, Volume of oxygen; ex, exercise; vs., Versus; SD, Standard deviation; IV, Inverse variance; CI, Confidence interval; HI, High-intensity; LI, Low-intensity).

In-patient CR, 6MWD was reported in five studies [33, 34, 39, 40, 43], and Peak ${\mathrm{VO}}_{2}$ in two studies [34, 43]. Manoeuvres involved active upper and lower limb exercises, breathing exercises, aerobic exercises, and IMT. Meanwhile, we found from an article that the IMT and breathing exercise group showed minor 6MWD improvement compared with the only breathing exercise group [43]. In particular, from one study [39] we found that in-patient triple exercise therapy contains hand gripe resistance training, aerobic and breathing exercise is insignificant to aerobic and breathing exercise therapy group for 6MWD improvement (Fig. 2). In contrast, In-patient peak ${\mathrm{VO}}_{2}$ for the quadruple exercise therapy group consisting of aerobic, resistance, balance, and calisthenics exercise showed insignificant results compared with aerobic and calisthenics as double exercise therapy at discharge. Still, patients in multiple exercise therapy with inspiratory muscle training, upper and lower limb exercise, and aerobic exercise showed significant improvement in peak ${\mathrm{VO}}_{2}$ during follow-up and 6MWD (Figs. 2,3). This improvement explains exercise principles such as overload (muscles needs to be trained higher level, which can increase the size of the cells and improve functional capacity) and specificity (exercise programs are designed to train specific muscle groups to meet metabolic demand and training response) [45]. Therefore, we conclude that in-patient CR with multiple exercise therapy (at minimum, double exercise therapy) is better than single exercise therapy for improving patient functional and exercise capacity.

The results of two studies [32, 38] involved out-patients CR on 6MWD and peak ${\mathrm{VO}}_{2}$. Our findings demonstrated that both outcomes were insignificant among patients with high-intensity IMT coupled with aerobic exercise to only aerobic exercise therapy (Figs. 2,3). On the other hand, the combination of moderate to high-intensity IMT, aerobic exercise, and resistance exercise as triple exercise therapy improve 6MWD and peak ${\mathrm{VO}}_{2}$ significantly with high certainty found in Grading of recommendations, assessment, development, and evaluations (GRADE) evidence at discharge compared to low-intensity exercise therapy (Table 2 and **Supplementary Table 5**). These findings suggest that to improve functional and exercise capacity, out-patient CR with high-intensity multiple exercise therapy is better than single exercise therapy.

**Table 2.** **GRADE quality assessment for 6MWD**.

Outcomes		Total number of participants (No. of study)	GRADE Certainty of the evidence ^a^	Anticipated absolute effects (95% CI) ^b^	
				Mean value in the control group	Std. Mean difference in Intervention group with control group
Functional capacity assessed with 6MWD					
In-patient	Multiple exercises. Vs. single exercise (discharge)	119 (2)	⊕⊕⊕◯	325.4 m	0.75; 95% CI (0.22, 1.28)
			MODERATE ^c,d,e^		
	Triple exercises Vs. double exercise (discharge)	15 (1)	⊕⊕⊕◯	318 m	–0.37; 95% CI (–1.25, 0.51)
			MODERATE ^c,d,e^		
	Multiple exercises Vs. double exercise (discharge)	191 (2)	⊕⊕◯⃝	247.4 m	0.41; 95% CI (–0.22, 1.03)
			LOW ^c,e^		
	Multiple exercises. Vs. single exercise (Follow-up)	128 (2)	⊕⊕⊕◯	458.6 m	0.85; 95% CI (0.26, 1.44)
			MODERATE ^c,e^		
Out-patient (discharge)	Double exercises Vs. single exercise	18 (1)	⊕⊕◯⃝	620 m	0.21; 95% CI (–0.72, 1.14)
			LOW ^c^		
	HI triple exercises Vs. LI triple exercise	24 (1)	⊕ ⊕ ⊕ ⊕	459.1 m	1.39; 95% CI (0.48, 2.29)
			HIGH ^f^		

^a^ GRADE (**G**rading of **R**ecommendations, **A**ssessment, **D**evelopment, and **E**valuations) working group for grading of trial evidence. High certainty refers to a high confident result where the true value is relatively close to that of the estimate of the effect. Moderate certainty refers to moderate confidence in the effect estimate, the true value is seeming to be close to the estimated effect with a probable difference. Low certainty refers to less confidence due to limited effect and substantial difference in the estimated effect. Very low certainty refers to very less confidence in effect because the true effect seems to be substantially different from the estimated effect. ^b^ the risk in the intervention group and its 95% confidence interval [CI] and fixed effect was used to measure risk in the comparison group. ^c^ Substantial problem finds in outcome measurement, missing outcome data, and reporting bias. ^d^ Risk with randomization of subjects. ^e^ Considerable heterogeneity found. ^f^ Did not find any problem with the risk of bias and sample size was considerable, high confidence of interval found. 6MWD, six-minute walking distance; m, meter; HI, High-intensity; LI, Low-intensity; ⊕/○, indicator of certainty of GRADE evidence (high, moderate, low, and very low).

#### 3.4.2 Health-Related QoL

Three studies [32, 33, 40] reported QoL results using the SF-36 questionnaire. Author Hirschhorn and colleagues [33] reported a significant improvement after in-patient CR at discharge among the single exercise group on domain vitality and the double exercise group on domain bodily pain. However, an insignificant result was found in the same domain after in-patient CR at follow-up and out-patient CR at discharge [33]. Another study on SF-36 among in-patient CR with multiple exercise groups also found insignificant improvement (*p *> 0.05) [40]. All other domains were found statistically insignificant after multiple exercises, both in-patient and out-patient CR. One study [38] using MLHFQ of the Portuguese version demonstrated that out-patient CR with double exercise group has a better outcome than the triple exercise group. In contrast with in-patient CR in the MacNew questionnaire of HRQL from one study [34] focusing on 4-domains (emotional, physical, social, and global), double exercise has a significant outcome compared to triple exercise groups. To sum up, more than double exercise therapy may have insignificant results for improving the QoL for CABG patients.

### 3.5 Risk of Bias and Quality Assessment

**Fig. 4.**
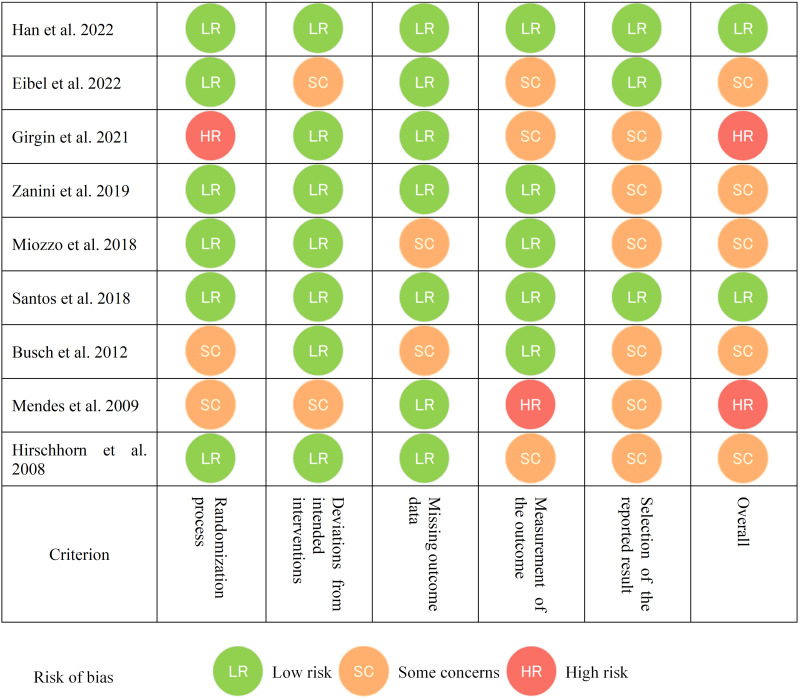
**Risk of bias among study**. This figure shows risk of bias according to the RoB 2 tool for each included study.

The results of risk of bias using the RoB 2 tool (https://www.riskofbias.info) in included studies noted the following: two studies [34, 42] had some concern about randomization, and one article had high risk [40] due to the involvement of physical therapists and other medical staff; two studies [32, 34] had some concern about missing outcome data because subjects missed follow-up analysis schedule; one study [42] had a high risk of outcome measurement because the accessor was concerned about the protocol; another three studies [33, 39, 40] showed some concern because of data accessors involvement. Finally, regarding the selection of study reporting, three studies [38, 39, 41] exhibited a low risk, whereas the remaining six studies [32, 33, 34, 40, 42, 43] had some concerns (Fig. 4).

In addition, the results of the PEDro scale from four studies [32, 38, 41, 43] showed scores from 10 to 9, considered a high-quality study. In contrast, the remaining studies [33, 34, 39, 40, 42] scored from 8 to 6, equivalent to a good quality study, as described previously (**Supplementary Table 6**).

## 4. Discussion

This systematic review focused exclusively on the number and combination of exercises in a protocol used for CABG patients in CR, which makes it different from other reviews. We found that a rehabilitation protocol with inspiratory muscle training, breathing, and aerobic exercise coupled with either upper or lower limb exercise is noticeably more effective for enhancing functional and exercise capacity. Some studies utilized inspiratory muscle training as high or moderate to high intensity. The combination of high-intensity IMT, aerobic, and resistance exercise seems beneficial regardless of age. Further, resistance exercise with balance and aerobic exercise has a more significant effect than only aerobic exercise among patients over 75 years of age (Table 1). Given the combinations, concerns arise regarding the choice of a treatment plan, rehabilitation cost, and time for the CABG population. Our review reveals that a specific exercise combination as a multiple exercise protocol is essential, and double or triple exercise therapy should be considered in clinical practice after evaluating the available treatment facility and patient capability.

Exercise is planned, purposeful, and repetitive movement that aims to gain cardiorespiratory and muscular endurance, strength, and flexibility [46]. A moderate level of exercise affects arterial blood vessels and enhances vasodilation and endothelial nitric oxide synthesis among stable CAD patients [47]. A meta-analysis of RCT on hypertensive patients found that more than half an hour of exercise, weekly 3 to 5 times above 80% of the maximal capacity of individuals, reduce blood pressure significantly compared to low-intensity exercise [48]. Further, exercise relieves musculoskeletal pain and improves functional capacity among patients with coronary artery bypass graft surgery by saphenous vein [43]. Hansen and colleagues noted that low-intensity resistance training with aerobic exercise improved plasma HDL and lean muscle mass (*p *< 0.05) and proved effective through improvement and reduction of functional capacity and length of hospitalization, respectively [25, 49]. In addition, a recent meta-analysis by Yamamoto *et al*. [50] found that resistance training with aerobic exercise increases peak ${\mathrm{VO}}_{2}$ (MD, 0.70 mL/kg/min; 95% CI, 0.03–1.37) and improves muscle strength for middle-aged and elderly patients with CAD. Our study found moderate to high-intensity IMT with aerobic and resistance training in out-patient CR improved functional and exercise capacity. Evidence suggests that this combined exercise is beneficial and safer for cardiac patients [51]. CABG patients exposed to multiple exercises showed significant improvement in functional capacity at hospital discharge immediately after/or 30-day, enhancing oxygen uptake and restoring extravascular fluid and mobility [52]. A recent meta-analysis emphasizes exercise-based CR protocol for chronic heart disease patients to reduce hospitalization, and mortality, and improve QoL [53].

To our knowledge, the effect of multiple exercises on only CABG patients in different measures against a single exercise therapy through systematic review was unrevealed. For instance, the 6MWD is widely accepted for measuring exercise tolerance and functional capacity ≥300 m average distance covered in 6MWD by the age of ≥65 is associated with less mortality [54, 55]. In our findings, multiple exercise therapy group patients had better results during in-patient CR; 6MWD increased in triple and double exercise groups compared to single exercise therapy. Further, when compared triple against double exercise, 6MWD during out-patient CR, triple exercise exhibited superior results (Table 2). In addition, for patients with multiple morbidities, exercise positively affects forced expiratory volume, forced vital capacity, maximum inspiratory pressure, and Borg exertion scores, as evidenced by a retrospective study by Liu and colleagues [56].

Numerous studies illustrated that an exercise program combined with early mobilization and strengthening training reduces the chances of emboli deposition inside vessels and increases the patient’s range of motion and activity in daily living. In addition, cardiac rehabilitation with exercise improves patients’ peak ${\mathrm{VO}}_{2}$ levels, which is a prognosticator for cardiac fitness and mortality [57]. Our study observed that patients in in-patient and out-patient CR exposed to multiple exercise therapy significantly progressed in peak ${\mathrm{VO}}_{2}$ more than in the single exercise therapy group.

Cardiac patients seem to have low health-related QoL before and after surgery, and SF-36 is the most used tool to assess patient QoL before and after rehabilitation [58]. Notably, we found that additional exercises to CR protocol slightly improved the SF-36 QoL questionnaire. Other measurement tools, such as MLHFQ of the Portuguese version and the MacNew questionnaire of HRQL, showed significant results in double exercise therapy groups than in triple exercise therapy groups. Therefore, studies support double and triple exercise therapy as an effective exercise protocol. However, further studies on treatment frequency, intensity, and time are required to consider these findings in clinical practice.

Moreover, our findings illustrated that patient-reported causes of CR drop-out were general health conditions, excessive demand for treatment outcomes, hospitals, and available transport facilities. Several reports stated that participation in CR is low among all eligible patients. In addition, we did not find any reported correlation between exercise choice and patient participation with drop-out among all included studies. Home-based cardiac rehabilitation with minimal education and proper guidelines on exercise can improve patient health and participation in CR [59, 60]. A recent systematic review comprised nine RCTs that included home-based CR and monitored patients’ exercise regimens by wearable smart devices, real-time calls, and nurses’ home visits. This review found a very low number of cardiovascular-related adverse events (e.g., hypotensive/hypertensive response) following home-based CR. Notably, no deaths were reported as a result of exercise training, albeit the authors advised taking extra precautions during the first session of the rehabilitation program [61]. A Cochrane study also suggests that home-based cardiac rehabilitation is safe for CABG patients [62]. However, one RCT found that center-based CR improves QoL more than home-based CR [63]. Nevertheless, an RCT on home-based CR protocol concerning multiple exercise interventions among the CABG population in a large cohort is necessary for implementing our findings. Likewise, our study proposes that a post-CABG cardiac rehabilitation protocol that contains double-exercise therapy is more worthwhile than single-exercise therapy.

We observed that exercise combinations differed in multiple exercise groups. Most studies explored multiple exercise programs as combining aerobic exercise, IMT, and resistance training, compared it with a single exercise program, such as breathing or aerobic exercise, and found significant improvement in multiple exercise groups [32, 34, 38, 39]. Considerably, any exercise paired with high-intensity or moderate to high-intensity IMT shows a statistically significant effect over other multiple exercise programs (combination of aerobic exercise and limb exercise) [32, 38]. Our findings suggest that a multiple exercise program comprising high-intensity IMT, aerobic exercise, and resistance training in both in-patient and out-patient CR settings (one time/day) may benefit CABG patients’ in enhancing functional capacity, exercise capacity, and QoL.

### Clinical Implementation, Limitations, and Recommendations

Evidence on only CABG rehabilitation through exercise therapy is deficient. Our study exhibited a few limitations; we included articles on only CABG patients, reducing article numbers and specific data. Due to insufficient data, we could not categorize studies according to exercise frequency, intensity, time, and training, which caused high heterogeneity, and sub-group analysis was also unplausible. We presented a forest plot and narrative synthesis of outcomes to make our findings more constructive. Further, our finding only demonstrated the exercise number without considering the treatment frequency and duration because each study has a different frequency and duration. These findings support the modification of exercise guidelines in light of CABG patients’ perspectives on exercise. Although our study provides comparative evidence on the effect of different exercise combinations from the last two decades, these findings will yield the development of a standard multiple-exercise protocol for CABG patients. For robustness, a future meta-analysis needs to include similar exercises as the multiple exercise group with the same treatment frequency intensity, time, and type. According to our findings, double and triple exercise therapy for CABG patients in a rehabilitation protocol is important, but further research is necessary to find a standard maximum exercise number for CABG patients. Hereafter, an RCT with a large cohort of CABG patients in CR comparing age and multiple exercise effects is warranted.

## 5. Conclusions

Our review suggests that, unlike single exercise, a combination of limb exercises, inspiratory muscle training, and aerobic and resistance exercise with proper supervision, as multiple exercise therapy for in-patient and out-patient cardiac rehabilitation, improves functional and exercise capacity. In contrast, multiple rehabilitative exercises showed insignificant results on health-related QoL among CABG patients. Further study on the effect of multiple exercises on functional capacity, Peak ${\mathrm{VO}}_{2}$, and QoL in a larger cohort with known vessel graft numbers is required.

## Abbreviations

CABG, coronary artery bypass graft; CR, cardiac rehabilitation; 6MWD, 6-minute walking distance; ${\mathrm{VO}}_{2}$, volume of oxygen; QoL, quality of life; IMT, inspiratory muscle training; MLHFQ, minnesota living with heart failure questionnaire; HRQL, health-related quality of life; HDL, high-density lipoprotein; RCT, randomized control trial; SWiM, synthesis without meta-analysis; SF-36, short form 36; MD, mean difference; PRISMA, preferred reporting items for systematic reviews and meta-analyses; GRADE, Grading of Recommendations, Assessment, Development, and Evaluations.

## Registration

This systematic review protocol has been registered to PROSPERO (Reg. No. CRD42021259327).

## Supplementary Material

Supplementary material associated with this article can be found, in the online version, at https://doi.org/10.31083/j.rcm2405141.
