# Supplementary material for: Efficacy of Multiple Exercise Therapy after Coronary Artery Bypass Graft: A Systematic Review of Randomized Control Trials
Source: Rev Cardiovasc Med. 2023 May 9;24(5):141. doi: 10.31083/j.rcm2405141 (PMC11273060; doi:10.31083/j.rcm2405141)
Supplement: Supplementary file 1 [file 2153-8174-24-5-141-s1.zip › 2153-8174-24-5-141-s1.docx]

*Supplementary Online Content*

Supplementary Method 1: The PRISMA checklist.

Supplementary Method 2: The SWiM checklist.

Supplementary Method 3: Studies on hand searched.

Supplementary Method 4: Unpublished numerical data from Miozzo et al. 2018.

Supplementary Method 5: Unpublished numerical data from Hirschhorn et al. 2008.

Supplementary Method 6: Error data from Busch et al. 2012.

Supplementary Table 1: Search strategy and result from all databases.

Supplementary Table 2: Baseline data among included articles.

Supplementary Table 3: Exercises grouping with a synthesis of outcome data on 6MWD and PeakVO_2_.

Supplementary Table 4: Exercise protocol for CABG patients among the included article.

Supplementary Table 5: GRADE quality assessment for Peak VO_2_.

Supplementary Table 6: PEDro scores for included studies.

Supplementary Method 1. **The PRISMA checklist.**

| **Section and Topic** | **Item #** | **Checklist item** | **Location where item is reported** |
| --- | --- | --- | --- |
| **TITLE** | | |  |
| Title | 1 | Identify the report as a systematic review. | Checked |
| **ABSTRACT** | | |  |
| Abstract | 2 | See the PRISMA 2020 for Abstracts checklist. | Checked Abstract |
| **INTRODUCTION** | | |  |
| Rationale | 3 | Describe the rationale for the review in the context of existing knowledge. | 1^st^ to 3^rd^ paragraph |
| Objectives | 4 | Provide an explicit statement of the objective(s) or question(s) the review addresses. | Last paragraph |
| **METHODS** | | |  |
| Eligibility criteria | 5 | Specify the inclusion and exclusion criteria for the review and how studies were grouped for the syntheses. | 1^st^ Paragraph |
| Information sources | 6 | Specify all databases, registers, websites, organisations, reference lists and other sources searched or consulted to identify studies. Specify the date when each source was last searched or consulted. | Search strategy 1^st^ para |
| Search strategy | 7 | Present the full search strategies for all databases, registers and websites, including any filters and limits used. | Flowchart 1 and Methods S1 in the supplemental |
| Selection process | 8 | Specify the methods used to decide whether a study met the inclusion criteria of the review, including how many reviewers screened each record and each report retrieved, whether they worked independently, and if applicable, details of automation tools used in the process. | Study selection 1^st^ paragraph |
| Data collection process | 9 | Specify the methods used to collect data from reports, including how many reviewers collected data from each report, whether they worked independently, any processes for obtaining or confirming data from study investigators, and if applicable, details of automation tools used in the process. | Data collection 1^st^ paragraph |
| Data items | 10a | List and define all outcomes for which data were sought. Specify whether all results that were compatible with each outcome domain in each study were sought (e.g. for all measures, time points, analyses), and if not, the methods used to decide which results to collect. | Data collection 2^nd^ paragraph |
|  | 10b | List and define all other variables for which data were sought (e.g. participant and intervention characteristics, funding sources). Describe any assumptions made about any missing or unclear information. | Data collection 2^nd^ paragraph |
| Study risk of bias assessment | 11 | Specify the methods used to assess risk of bias in the included studies, including details of the tool(s) used, how many reviewers assessed each study and whether they worked independently, and if applicable, details of automation tools used in the process. | Study risk of bias 1^st^ paragraph |
| Effect measures | 12 | Specify for each outcome the effect measure(s) (e.g. risk ratio, mean difference) used in the synthesis or presentation of results. | Data synthesis 1^st^ paragraph |
| Synthesis methods | 13a | Describe the processes used to decide which studies were eligible for each synthesis (e.g. tabulating the study intervention characteristics and comparing against the planned groups for each synthesis (item #5)). | Table 1 |
|  | 13b | Describe any methods required to prepare the data for presentation or synthesis, such as handling of missing summary statistics, or data conversions. | Data collection last sentence, Methods S4-S6 in the supplemental |
|  | 13c | Describe any methods used to tabulate or visually display results of individual studies and syntheses. | Data synthesis 1^st^ sentence |
|  | 13d | Describe any methods used to synthesize results and provide a rationale for the choice(s). If meta-analysis was performed, describe the model(s), method(s) to identify the presence and extent of statistical heterogeneity, and software package(s) used. | Data synthesis 2^nd^ sentence |
|  | 13e | Describe any methods used to explore possible causes of heterogeneity among study results (e.g. subgroup analysis, meta-regression). | Data synthesis last sentence |
|  | 13f | Describe any sensitivity analyses conducted to assess robustness of the synthesized results. | Data synthesis last sentence |
| Reporting bias assessment | 14 | Describe any methods used to assess risk of bias due to missing results in a synthesis (arising from reporting biases). | Risk of bias assessment 1^st^ and 2^nd^ sentence |
| Certainty assessment | 15 | Describe any methods used to assess certainty (or confidence) in the body of evidence for an outcome. | Risk of bias assessment 2^nd^ sentence |
| **RESULTS** | | |  |
| Study selection | 16a | Describe the results of the search and selection process, from the number of records identified in the search to the number of studies included in the review, ideally using a flow diagram. | Search results |
|  | 16b | Cite studies that might appear to meet the inclusion criteria, but which were excluded, and explain why they were excluded. | Search results |
| Study characteristics | 17 | Cite each included study and present its characteristics. | Study characteristics 1^st^ paragraph |
| Risk of bias in studies | 18 | Present assessments of risk of bias for each included study. | Table 5 in the supplemental and fig. 4 |
| Results of individual studies | 19 | For all outcomes, present, for each study: (a) summary statistics for each group (where appropriate) and (b) an effect estimate and its precision (e.g. confidence/credible interval), ideally using structured tables or plots. | Result of outcomes paragraphs |
| Results of syntheses | 20a | For each synthesis, briefly summarise the characteristics and risk of bias among contributing studies. | Risk of bias paragraph |
|  | 20b | Present results of all statistical syntheses conducted. If meta-analysis was done, present for each the summary estimate and its precision (e.g. confidence/credible interval) and measures of statistical heterogeneity. If comparing groups, describe the direction of the effect. | Results of outcomes |
|  | 20c | Present results of all investigations of possible causes of heterogeneity among study results. | Checked |
|  | 20d | Present results of all sensitivity analyses conducted to assess the robustness of the synthesized results. | Checked |
| Reporting biases | 21 | Present assessments of risk of bias due to missing results (arising from reporting biases) for each synthesis assessed. | Checked |
| Certainty of evidence | 22 | Present assessments of certainty (or confidence) in the body of evidence for each outcome assessed. | Table 1 and 2 |
| **DISCUSSION** | | |  |
| Discussion | 23a | Provide a general interpretation of the results in the context of other evidence. | 1^st^ paragraph |
|  | 23b | Discuss any limitations of the evidence included in the review. | Limitation paragraph |
|  | 23c | Discuss any limitations of the review processes used. | Limitation paragraph |
|  | 23d | Discuss implications of the results for practice, policy, and future research. | Conclusion paragraph |
| **OTHER INFORMATION** | | |  |
| Registration and protocol | 24a | Provide registration information for the review, including register name and registration number, or state that the review was not registered. | Other information |
|  | 24b | Indicate where the review protocol can be accessed, or state that a protocol was not prepared. | checked |
|  | 24c | Describe and explain any amendments to information provided at registration or in the protocol. | checked |
| Support | 25 | Describe sources of financial or non-financial support for the review, and the role of the funders or sponsors in the review. | checked |
| Competing interests | 26 | Declare any competing interests of review authors. | checked |
| Availability of data, code and other materials | 27 | Report which of the following are publicly available and where they can be found: template data collection forms; data extracted from included studies; data used for all analyses; analytic code; any other materials used in the review. | Supplemental file provided |

From: Page MJ, McKenzie JE, Bossuyt PM, Boutron I, Hoffmann TC, Mulrow CD, et al. The PRISMA 2020 statement: an updated guideline for reporting systematic reviews. BMJ 2021;372:n71. doi: 10.1136/bmj.n71

Supplementary Method 2. The **SWiM checklist.**

| **SWiM reporting item** | **Item description** | **Section in manuscript where item is reported** |
| --- | --- | --- |
| **Methods** | | |
| 1 Grouping studies for synthesis | 1a) Provide a description of, and rationale for, the groups used in the synthesis (eg, groupings of populations, interventions, outcomes, study design) | Data Collection and synthesis |
|  | 1b) Detail and provide rationale for any changes made subsequent to the protocol in the groups used in the synthesis | Data Collection and synthesis |
| 2 Describe the standardised metric and transformation methods used | Describe the standardised metric for each outcome. Explain why the metric(s) was chosen and describe any methods used to transform the intervention effects, as reported in the study, to the standardised metric, citing any methodological guidance consulted | Data Collection and synthesis |
| 3 Describe the synthesis methods | Describe and justify the methods used to synthesise the effects for each outcome when it was not possible to undertake a meta-analysis of effect estimates | Data Collection and synthesis |
| 4 Criteria used to prioritise results for summary and synthesis | Where applicable, provide the criteria used, with supporting justification, to select the particular studies, or a particular study, for the main synthesis or to draw conclusions from the synthesis (eg, based on study design, risk of bias assessments, directness in relation to the review question) | Data Collection and synthesis |
| 5 Investigation of heterogeneity in reported effects | State the method(s) used to examine heterogeneity in reported effects when it was not possible to undertake a meta-analysis of effect estimates and its extensions to investigate heterogeneity | Data Collection and synthesis |
| 6 Certainty of evidence | Describe the methods used to assess the certainty of the synthesis findings | Data Collection and synthesis |
| 7 Data presentation methods | Describe the graphical and tabular methods used to present the effects (eg, tables, forest plots, harvest plots)  Specify key study characteristics (eg, study design, risk of bias) used to order the studies, in the text and any tables or graphs, clearly referencing the studies included | Data Collection and synthesis |
| **Results** | | |
| 8 Reporting results | For each comparison and outcome, provide a description of the synthesised findings and the certainty of the findings. Describe the result in language that is consistent with the question the synthesis addresses, and indicate which studies contribute to the synthesis | Result |
| **Discussion** | | |
| 9 Limitations of the synthesis | Report the limitations of the synthesis methods used and/or the groupings used in the synthesis and how these affect the conclusions that can be drawn in relation to the original review question | Clinical Implementation, limitations, and recommendations |

From: Campbell M, McKenzie JE, Sowden A, et al. Synthesis without meta-analysis (SWiM) in systematic reviews: reporting guideline. *BMJ*. 2020;368:l6890. doi:10.1136/bmj.l6890

Supplementary Method [3](#eMethods2). Studies on hand searched.

1. Liu J-F, Lee H-M, Chen J-O, et al. Benefits of Multiple-Intervention Pulmonary Rehabilitation to Older Adults with High-Risk Multimorbidity after Coronary Artery Bypass Grafting. *Healthcare*. 2020;8(4):368. doi:10.3390/healthcare8040368

2. Westerdahl E, Fagevik Olsén M. Chest physiotherapy and breathing exercises for cardiac surgery patients in Sweden - A National survey of practice. *Monaldi Arch Chest Dis*. 2015;75(2). doi:10.4081/monaldi.2011.223

3. Fitchet A. Comprehensive cardiac rehabilitation programme for implantable cardioverter-defibrillator patients: a randomized controlled trial. *Heart*. 2003;89(2):155-160. doi:10.1136/heart.89.2.155

4. Brasher PA, McClelland KH, Denehy L, Story I. Does removal of deep breathing exercises from a physiotherapy program including pre-operative education and early mobilization after cardiac surgery alter patient outcomes? *Australian Journal of Physiotherapy*. 2003;49(3):165-173. doi:10.1016/S0004-9514(14)60236-1

5. Osailan A, Abdelbasset WK. Exercise-based cardiac rehabilitation for post coronary artery bypass grafting: its effect on hemodynamics response and functional capacity using Incremental Shuttle walking test: a retrospective pilot analysis. *Journal of the Saudi Heart Association*. 2020;32(1). doi:10.37616/2212-5043.1005

6. Filbay SR, Hayes, K, Holland, AE. Physiotherapy for patients following coronary artery bypass graft (CABG) surgery: Limited uptake of evidence into practice. *Physiotherapy Theory and Practice*. 2012;28(3):178-187. doi:10.3109/09593985.2011.582231

7. Hulzebos EHJ, Helders PJM, Favié NJ, De Bie RA, Brutel de la Riviere A, Van Meeteren NLU. Preoperative Intensive Inspiratory Muscle Training to Prevent Postoperative Pulmonary Complications in High-Risk Patients Undergoing CABG Surgery: A Randomized Clinical Trial. *JAMA*. 2006;296(15):1851. doi:10.1001/jama.296.15.1851

8. Cacciatore F, Abete P, Mazzella F, et al. Six-minute walking test but not ejection fraction predicts mortality in elderly patients undergoing cardiac rehabilitation following coronary artery bypass grafting. *Eur J Prev Cardiolog*. 2012;19(6):1401-1409. doi:10.1177/1741826711422991

Supplementary Method 4. Unpublished numerical data from Miozzo et al. 2018.

In the published article, Miozzo et al. did not mention the number of subjects according to Male and Female and 6MWD data on the numerical value of Mean and Standard deviation (SD). We contacted through the ResearchGate platform and collected data.

Table of unpublished data for gender:

| **Group** | **Male, n (%)** | **Female, n (%)** | **Total** |
| --- | --- | --- | --- |
| GAE+IMT | 7(77.7) | 2(22.3) | 9 |
| GAE | 8(88.9) | 1(11.2) | 9 |

GAE (Aerobic exercise group), IMT (Inspiratory muscle training group)

Table of unpublished data 6-minute walking test:

| 6MWT(m) | GAE+IMT | GAE |
| --- | --- | --- |
| Pre-intervention | 543.3 (111) | 545.5 (161) |
| Discharge | 638.6 ( 84) | 620 (85.6) |

GAE (Aerobic exercise group), IMT (Inspiratory muscle training group), 6MWT (Six-minute walking test); Data presented in Mean and Standard Deviation (SD)

| **MIP** | time 0 | time 12 | time 24 | time 36 |
| --- | --- | --- | --- | --- |
| GAE + IMT | 81 (29.11) | 101(33.35) | 132(29.11) | 128(29.51) |
| GAE | 81.7(26.4) | 88.3(28.3) | 98.3(25.1) | 101.1(28.20) |

GAE (Aerobic exercise group), MIP (Maximum Inspiratory Pressure), 6MWT (Six-minute walking test); Data presented in Mean and Standard Deviation (SD)

| **MEP** | time 0 | time 12 | time 24 | time 36 |
| --- | --- | --- | --- | --- |
| GAE + IMT | 106(31.39) | 142(34.86) | 157(32.96) | 163(33.72) |
| GAE | 114(37.3) | 124(34.3) | 127(37) | 130(39) |

GAE (Aerobic exercise group), MEP (Maximum Expiratory Pressure), 6MWT (Six-minute walking test); Data presented in Mean and Standard Deviation (SD)

From: Miozzo AP, Stein C, Marcolino MZ, et al. Effects of high-intensity inspiratory muscle training associated with aerobic exercise in patients undergoing CABG: Randomized clinical trial. *Brazilian Journal of Cardiovascular Surgery*. 2018;33(4):376-383.

Supplementary Method 5. Unpublished numerical data from Hirschhorn et al. 2008.

Unpublished data for 6-minute walking Assessment (6MWA) was collected from author Hirschhorn et al. through his ResearchGate account as a picture.

Table of unpublished data: Mean and Standard Deviation (SD)

| **6MWA(m)** | **Standard Intervention; mean (SD)** | **Participants, (n)** | **Walking (aerobic) exercise; mean (SD)** | **Participants, (n)** | **Walking (aerobic) and breathing exercise; mean (SD)** | **Participants, (n)** |
| --- | --- | --- | --- | --- | --- | --- |
| Pre-operative | 456 (98) | 24 | 476 (69) | 24 | 495(68) | 19 |
| Discharge | 377(90) | 30 | 444 (84) | 30 | 413(98) | 30 |
| Follow-up | 497(76) | 29 | 527(78) | 30 | 518(99) | 29 |

From: Hirschhorn AD, Richards D, Mungovan SF, Morris NR, Adams L. Supervised moderate intensity exercise improves distance walked at hospital discharge following coronary artery bypass graft surgery--a randomized controlled trial. *Heart Lung Circ*. 2008;17(2):129-138. doi:10.1016/j.hlc.2007.09.004

Supplementary Method 6. Error data from Busch et al. 2012.

A printing error on the Randomization population and distribution was corrected by the author via email as a PDF file which was shown in a poster presentation of this study.

Table of error data:

| Area | Previous or published | Corrected or Unpublished |
| --- | --- | --- |
| Accessed eligibility | 212 | 382 |
| Excluded | 111 | 209 |
| Randomized | 121 | 173 |
| Intervention group | 57 | 84 |
| Data analyzes | 107 | 150 |
| Male | IG, 28; CG, 26 | IG, 56; CG, 63 |
| Female | IG, 56; CG, 63 | IG, 28; CG, 26 |

IG= Intervention group; CG= Control group

From: Busch JC, Lillou D, Wittig G, et al. Resistance and Balance Training Improves Functional Capacity in Very Old Participants Attending Cardiac Rehabilitation After Coronary Bypass Surgery. *Journal of the American Geriatrics Society*. 2012;60(12):2270-2276

Supplementary Table 1. Search strategy and result from all databases.

| **Search No** | **Search Term** | **Database, Advanced Search Mode, and Limiters** | **Reasons** | **Search Results**  15-09-2022 |
| --- | --- | --- | --- | --- |
| 1 | - Exercise - therap* - “Physical Activity” | EBSCOhost: Boolean/Phrase: OR, all fields, Full text, published date:20000101-20211231 | To identify the effect of different rehabilitation programs as an intervention and comparator. | 6,459,528 |
|  |  | Web of Science: Boolean/Phrase: OR, Topic Sentence, Date of publication:2000/2021, Document type: Article, All open access, Language: English or Chinese or Russian. |  | 3,082,624 |
|  |  | Scopus: TITLE-ABS-KEY, Boolean/Phrase: OR, Publication stage: Final, Document type: Article, Limit to publication year: 2000-2021, Language: English, Chinese, Russian |  | 1,387,459 |
|  |  | PubMed: All fields, Boolean/Phrase: OR, Publication date: 2000-2021 |  | [5,378,823](https://pubmed.ncbi.nlm.nih.gov/?term=%28%28Exercise%29+OR+%28therap%2A%29%29+OR+%28Physical+Activity%29&filter=years.2000-2022&sort=relevance) |
| 2 | - Physiotherapy - “Physical Therapy” - “Occupational Therapy” - Rehab* - Prehab* - postrehab* - Preoperative - Postoperative | EBSCOhost: Boolean/Phrase: OR, all fields, Full text, published date:20000101-20211231 | To find differenttypes of rehabilitation  Protocols’ outcomes. | 1,565,413 |
|  |  | Web of Science: Boolean/Phrase: OR, Topic Sentence, Date of publication:2000/2021, Document type: Article, All open access, Language: English or Chinese or Russian. |  | 481,462 |
|  |  | Scopus: TITLE-ABS-KEY, Boolean/Phrase: OR, Publication stage: Final, Document type: Article, Limit to publication year: 2000-2021, Language: English, Chinese, Russian |  | 344,388 |
|  |  | PubMed: All fields, Boolean/Phrase: OR, Publication date: 2000-2021 |  | [1,351,079](https://pubmed.ncbi.nlm.nih.gov/?term=%28%28%28%28%28%28%28Physiotherapy%29+OR+%28%22Physical+Therapy%22%29%29+OR+%28%22Occupational+Therapy%22%29%29+OR+%28Rehab%2A%29%29+OR+%28Prehab%2A%29%29+OR+%28postrehab%2A%29%29+OR+%28Preoperative%29%29+OR+%28Postoperative%29&filter=years.2000-2022&ac=no&sort=relevance) |
| 3 | - “Home based” - “Hospital based” | EBSCOhost: Boolean/Phrase: OR, all fields, Full text, published date:20000101-20211231 | To identity different rehabilitation phases as a comparator or Intervention | 101,402 |
|  |  | Web of Science: Boolean/Phrase: OR, Topic Sentence, Date of publication:2000/2021, Document type: Article, All open access, Language: English or Chinese or Russian. |  | 189,773 |
|  |  | Scopus: TITLE-ABS-KEY, Boolean/Phrase: OR, Publication stage: Final, Document type: Article, Limit to publication year: 2000-2021, Language: English, Chinese, Russian |  | 20,342 |
|  |  | PubMed: All fields, Boolean/Phrase: OR, Publication date: 2000-2021 |  | [41,111](https://pubmed.ncbi.nlm.nih.gov/?term=%28%22Home+based%22%29+OR+%28%22Hospital+based%22%29&filter=years.2000-2022&sort=relevance) |
| 4 | - “Patient participation” - Hospitaliz* | EBSCOhost: Boolean/Phrase: OR, all fields, Full text, published date:20000101-20211231 | To identify the cause and effect of a rehabilitation program. | 387,914 |
|  |  | Web of Science: Boolean/Phrase: OR, Topic Sentence, Date of publication:2000/2021, Document type: Article, All open access, Language: English or Chinese or Russian. |  | 151,341 |
|  |  | Scopus: TITLE-ABS-KEY, Boolean/Phrase: OR, Publication stage: Final, Document type: Article, Limit to publication year: 2000-2021, Language: English, Chinese, Russian |  | 172,237 |
|  |  | PubMed: All fields, Boolean/Phrase: OR, Publication date: 2000-2021 |  | [299,015](https://pubmed.ncbi.nlm.nih.gov/?term=%28%22Patient+participation%22%29+OR+%28Hospitaliz%2A%29&filter=years.2000-2022&sort=relevance) |
| 5 | COMBINE SEARCH 1-4 WITH “OR” | EBSCOhost: Boolean/Phrase: OR, S1 OR S2 OR S3 OR S4 | Combine all | 7,823,037 |
|  |  | Web of Science: Boolean/Phrase: OR, #1 OR #2 OR #3 OR #4 |  | [3,426,517](https://www.webofscience.com/wos/alldb/summary/1166a923-55ca-4551-ab02-21cccff7be40-4f1691dc/relevance/1) |
|  |  | Scopus: Boolean/Phrase: OR, #1 OR #2 OR #3 OR #4 |  | 1,735,937 |
|  |  | PubMed: Boolean/Phrase: OR, #1 OR #2 OR #3 OR #4 |  | [6,326,581](https://pubmed.ncbi.nlm.nih.gov/?term=%28%28%28%28%22Patient+participation%22%29+OR+%28Hospitaliz%2A%29+AND+%282000%3A2022%5Bpdat%5D%29%29+OR+%28%28%22Home+based%22%29+OR+%28%22Hospital+based%22%29+AND+%282000%3A2022%5Bpdat%5D%29%29%29+OR+%28%28%28Exercise%29+OR+%28therap%2A%29%29+OR+%28Physical+Activity%29+AND+%282000%3A2022%5Bpdat%5D%29%29%29+OR+%28%28%28%28%28%28%28%28Physiotherapy%29+OR+%28%22Physical+Therapy%22%29%29+OR+%28%22Occupational+Therapy%22%29%29+OR+%28Rehab%2A%29%29+OR+%28Prehab%2A%29%29+OR+%28postrehab%2A%29%29+OR+%28Preoperative%29%29+OR+%28Postoperative%29+AND+%282000%3A2022%5Bpdat%5D%29%29&ac=no&sort=relevance) |
| 6 | - Cardiac - cardiovascular - Heart - “Coronary artery” | EBSCOhost: Boolean/Phrase: OR, all fields, Full text, published date:20000101-20211231 | The specific article only focuses on cardiac diseases. | 3,089,738 |
|  |  | Web of Science: Boolean/Phrase: OR, Topic Sentence, Date of publication:2000/2021, Document type: Article, All open access, Language: English or Chinese or Russian. |  | 1,020,500 |
|  |  | Scopus: TITLE-ABS-KEY, Boolean/Phrase: OR, Publication stage: Final, Document type: Article, Limit to publication year: 2000-2021, Language: English, Chinese, Russian |  | 634,257 |
|  |  | PubMed: All fields, Boolean/Phrase: OR, Publication date: 2000-2021 |  |  |
| 7 | COMBINE SEARCH 5 & 6 WITH “AND” | EBSCOhost: Boolean/Phrase: AND, S5 AND S6 | Separate from other types of population | 864,366 |
|  |  | Web of Science: Boolean/Phrase: AND, #5 AND #6 |  | [586,377](https://www.webofscience.com/wos/alldb/summary/80b922e2-dbe5-42ff-9cb7-7b0b4af7adcf-4f16cbbe/relevance/1) |
|  |  | Scopus: Boolean/Phrase: AND, #5 AND #6 |  | 250,761 |
|  |  | PubMed: Boolean/Phrase: AND, #5 AND #6 |  | [907,642](https://pubmed.ncbi.nlm.nih.gov/?term=%28%28%28%28%28%22Patient+participation%22%29+OR+%28Hospitaliz%2A%29+AND+%282000%3A2022%5Bpdat%5D%29%29+OR+%28%28%22Home+based%22%29+OR+%28%22Hospital+based%22%29+AND+%282000%3A2022%5Bpdat%5D%29%29%29+OR+%28%28%28Exercise%29+OR+%28therap%2A%29%29+OR+%28Physical+Activity%29+AND+%282000%3A2022%5Bpdat%5D%29%29%29+OR+%28%28%28%28%28%28%28%28Physiotherapy%29+OR+%28%22Physical+Therapy%22%29%29+OR+%28%22Occupational+Therapy%22%29%29+OR+%28Rehab%2A%29%29+OR+%28Prehab%2A%29%29+OR+%28postrehab%2A%29%29+OR+%28Preoperative%29%29+OR+%28Postoperative%29+AND+%282000%3A2022%5Bpdat%5D%29%29%29+AND+%28%28%28%28Cardiac%29+OR+%28cardiovascular%29%29+OR+%28Heart%29%29+OR+%28Coronary+artery%29+AND+%282000%3A2022%5Bpdat%5D%29%29&ac=no&sort=relevance) |
| 8 | - “Cardiac rehab*” - “cardiac exercise” - “cardiac therapy” - “cardiovascular rehab*” - “cardiovascular exercise” - “cardiovascular therapy” | EBSCOhost: Boolean/Phrase: OR, all fields, Full text, published date:20000101-20211231 | To find specific rehabilitation  protocol only focuses on cardiac patient. | 16,585 |
|  |  | Web of Science: Boolean/Phrase: OR, Topic Sentence, Date of publication:2000/2021, Document type: Article, All open access, Language: English or Chinese or Russian. |  | 4,854 |
|  |  | Scopus: TITLE-ABS-KEY, Boolean/Phrase: OR, Publication stage: Final, Document type: Article, Limit to publication year: 2000-2021, Language: English, Chinese, Russian |  | 2,999 |
|  |  | PubMed: All fields, Boolean/Phrase: OR, Publication date: 2000-2021 |  | 17,174 |
| 9 | COMBINE SEARCH 7 & 8 WITH “AND” | EBSCOhost: Boolean/Phrase: AND, S7 AND S8 | Re-search for any missing rehabilitation protocol | 15,583 |
|  |  | Web of Science: Boolean/Phrase: AND, #7 AND #8 |  | [4,854](https://www.webofscience.com/wos/alldb/summary/06d01b72-4147-4844-9a23-ae1c6b2e5e57-4f1710b9/relevance/1) |
|  |  | Scopus: Boolean/Phrase: AND, #7 AND #8 |  | 2,999 |
|  |  | PubMed: Boolean/Phrase: AND, #7 AND #8 |  | 17,174 |
| 10 | - CABG - “coronary artery bypass graft*” - “coronary artery bypass” | EBSCOhost: Boolean/Phrase: OR, all fields, Full text, published date:20000101-20211231 | Identify specific articles on the study population. | 64,529 |
|  |  | Web of Science: Boolean/Phrase: OR, Topic Sentence, Date of publication:2000/2021, Document type: Article, All open access, Language: English or Chinese or Russian. |  | 18,235 |
|  |  | Scopus: TITLE-ABS-KEY, Boolean/Phrase: OR, Publication stage: Final, Document type: Article, Limit to publication year: 2000-2021, Language: English or Chinese or Russian |  | 21,146 |
|  |  | PubMed: All fields, Boolean/Phrase: OR, Publication date: 2000-2021 |  | [48,242](https://pubmed.ncbi.nlm.nih.gov/?term=%28%28CABG%29+OR+%28%22coronary+artery+bypass+graft%2A%22%29%29+OR+%28%22coronary+artery+bypass%22%29&filter=years.2000-2022&sort=relevance) |
| 11 | COMBINE SEARCH 9 & 10 WITH “AND” | EBSCOhost: Boolean/Phrase: AND, combine search S9 AND S10, Full text, published date:20000101-20211231, Article type: Journal, Language: English or Chinese or Russian. | Separate other populations from CABG. | 664^a^ |
|  |  | Web of Science: Boolean/Phrase: AND, combine search #9 AND #10, Topic Sentence, Date of publication:2000/2021, Document type: Article, All open access, Language: English or Chinese or Russian. |  | [348](https://www.webofscience.com/wos/alldb/summary/d494f060-5156-47b4-bf5d-68877532d72c-4f173746/relevance/1) |
|  |  | Scopus: TITLE-ABS-KEY, Boolean/Phrase: combine search #9 AND #10, Publication stage: Final, Document type: Article, Limit to publication year: 2000-2021, Language: English, Chinese, Russian. |  | 357 |
|  |  | PubMed: All fields, Boolean/Phrase: combine search #9 AND #10, Publication date: 2000-2021, full text, Journal Article, Language: English or Chinese or Russian. |  | 667 |

^a^ At the last page after the automatic removal of some duplicate articles. **2036**

Supplementary Table 2. Baseline data among included articles.

| Variables | Result  mean (SD), n (%) | Number of articles (reference) |
| --- | --- | --- |
| Age (year) | 66.35(11.07) | 9(Han et al. 2022, Eibel et al. 2022, Girgin et al. 2021, Miozzo et al. 2018, Hirschhorn et al. 2008, Busch et al. 2012, Zanini et al. 2019, Mendes et al. 2009, Santos et al. 2018) |
| Male | 433 (73.95%) | 9(Han et al. 2022, Eibel et al. 2022, Girgin et al. 2021, Miozzo et al. 2018, Hirschhorn et al. 2008, Busch et al. 2012, Zanini et al. 2019, Mendes et al. 2009, Santos et al. 2018) |
| Body mass index (kg/m^2^) | 26.53 (4.75) | 7(Han et al. 2022, Miozzo et al. 2018, Hirschhorn et al. 2008, Busch et al. 2012, Zanini et al. 2019, Mendes et al. 2009, Santos et al. 2018) |
| Hypertension | 312 (76.09%) | 8 (Han et al. 2022, Eibel et al. 2022, Miozzo et al. 2018, Busch et al. 2012, Zanini et al. 2019, Santos et al. 2018) |
| Diabetes mellitus | 117 (29.84%) | 5 (Han et al. 2022, Eibel et al. 2022, Busch et al. 2012, Zanini et al. 2019, Santos et al. 2018) |
| Beta-blocker use | 200 (72.2%) | 5 (Eibel et al. 2022, Miozzo et al. 2018, Busch et al. 2012, Mendes et al. 2009, Santos et al. 2018) |
| Length of ICU stay (days) | 9.65(6.6) | 3 (Hirschhorn et al. 2008, Zanini et al. 2019, Mendes et al. 2009) |
| Surgery duration (minute) | 175.81(48.79) | 3 (Hirschhorn et al. 2008, Zanini et al. 2019, Mendes et al. 2009) |
| Drop-out from cardiac rehabilitation | 62 (9.8%) | 9(Han et al. 2022, Eibel et al. 2022, Girgin et al. 2021, Miozzo et al. 2018, Hirschhorn et al. 2008, Busch et al. 2012, Zanini et al. 2019, Mendes et al. 2009, Santos et al. 2018) |
| Declined to participate | 144 (10.84%) | 6 (Miozzo et al. 2018, Hirschhorn et al. 2008, Busch et al. 2012, Zanini et al. 2019, Mendes et al. 2009, Santos et al. 2018) |

Supplementary Table 3. Exercises grouping with a synthesis of outcome data on 6MWD and PeakVO_2_.

| **Study, Sittings** | **Exercise-group** | **Outcome** | **Intervention** | **Intervention Group(s) post-intervention; Mean (SD), N** | **Control** | **Control group post-intervention;**  **Mean (SD), N** | **Study completion, (%)** | **Effect estimate;**  **OR** | **Conflict of Interest** |
| --- | --- | --- | --- | --- | --- | --- | --- | --- | --- |
| Han et al. (2022), In-patient | Triple exercise therapy vs double exercise therapy | Not available | Early mobilization, Aerobic and breathing exercises (triple exercise therapy) | Not available | Aerobic and breathing exercise (double exercise therapy) | Not available | 93.30 % | Not available | None |
| Eibel et al. (2022), In-patient | Triple exercise therapy vs. double exercise therapy (Eibel et al. 2022_A) | 6MWD  (discharge) | Resistance, aerobic and breathing exercise (triple exercise therapy) | 300.75 (26.29), 4 | Aerobic and breathing exercises (double exercise therapy) | 318(31.08), 6 | 100% | -0.96 | None |
|  | Triple exercise therapy vs. double exercise therapy (Eibel et al. 2022_B) | 6MWD  (discharge) | Inspiratory muscle training, aerobic and breathing exercise (triple Exercise therapy) | 306.2 (61.63), 5 | Aerobic and breathing exercises (double exercise therapy) | 318(31.08), 6 |  | -0.42 |  |
| Girgin et al. (2021), In-patient | Triple exercise therapy vs. double exercise therapy | 6MWD  (discharge) | Lower-upper extremity exercises, aerobic and breathing exercises (triple exercise therapy) | 177.8(58.5), 25 | Aerobic and breathing exercises (double exercise therapy) | 137.4(43.2), 25 | 100% | 1.40 | None |
| Zanini et al. (2019), In-patient | Double exercise therapy vs. single exercise therapy (Zanini et al. 2019_A) | 6MWD (discharge) | Inspiratory muscle training (IMT) and breathing exercises (double  exercise therapy) | 275.0 (69.57), 10 | Breathing exercise (single exercise therapy) | 291.00 (53.6), 10 | 100% (discharge)  97.5% (follow-up) | -0.45 | None |
|  |  | 6MWD (Follow-up) |  | 471.0(44.27), 10 |  | 433.0(44.27), 10 |  | 1.49 |  |
|  |  | Peak VO_2_ (Follow-up) |  | 17.7(3.2), 10 |  | 17.3(3.2), 9 |  | 0.22 |  |
|  | Double exercise therapy vs. single exercise therapy (Zanini et al. 2019_B) | 6MWD  (discharge) | Active UL and LL exercise training and breathing exercise (triple exercise  therapy) | 401.0(63.24), 10 | Breathing exercise (single  exercise therapy) | 291.00 (53.6), 10 |  | 3.26 |  |
|  |  | 6MWD (Follow-up) |  | 531.0(50.59), 10 |  | 433.0(44.27), 10 |  | 3.57 |  |
|  |  | Peak VO_2_ (Follow-up) |  | 21.4(2.8), 10 |  | 17.3(3.2), 9 |  | 2.38 |  |
|  | Triple exercise therapy vs. single exercise therapy (Zanini et al. 2019_C) | 6MWD  (discharge) | Inspiratory muscle training, active UL and LL exercise training, early mobilization and breathing exercise (triple  exercise therapy) | 365.0(66.41), 10 | Breathing exercise (single exercise therapy) | 291.00 (53.6), 10 |  | 2.12 |  |
|  |  | 6MWD (Follow-up) |  | 531.0(72.73), 10 |  | 433.0(44.27), 10 |  | 2.83 |  |
|  |  | Peak VO_2_ (Follow-up) |  | 21.4(3.1), 10 |  | 17.3(3.2), 9 |  | 2.25 |  |
| Mendes et al. (2009), In-patient | Triple exercise therapy vs. single exercise therapy | Not available | Supervised active-assistive exercises of UL and LL, breathing exercises, and ambulation (triple exercise therapy) | Not available | Breathing exercise (single exercise therapy) | Not available | 63.51% | Not available | Not available |
| Busch et al. (2012), In-patient | Quadruple exercise therapy vs. double exercise therapy | 6MWD  (discharge) | Aerobic exercise, calisthenics exercise, resistance training upper and lower limbs, balance training (quadruple exercise therapy) | 363.0(86.0), 68 | Aerobic exercise and calisthenics exercise (double exercise therapy) | 352.0(82.0), 73 | 88.42% | 0.23 | None |
|  |  | Peak VO_2_ (discharge) |  | 13.5(2.9),61 |  | 13.6(3.3), 65 |  | -0.05 |  |
| Hirschhorn et al. (2008), In-patient | Single exercise therapy vs. single exercise therapy (Hirschhorn et al. 2008_A) | 6MWD  (discharge) | Aerobic exercise with modified aerobic exercise  training (single exercise therapy) | 431.0(98.0), 19 | Ambulation (single exercise therapy) | 377.0(90.0), 30 | 97.85% (discharge)  96.70% (follow-up) | 1.03 | Not available |
|  |  | 6MWD (Follow-up) |  | 518.0(99.0), 29 |  | 497.0(76.0), 29 |  | 0.42 |  |
|  | Double exercise therapy vs. single exercise therapy (Hirschhorn et al. 2008_B) | 6MWD  (discharge) | Aerobic exercise and breathing exercise (double  exercise therapy) | 444.0(84.0), 30 | Ambulation (single exercise therapy) | 377.0(90.0), 30 |  | 1.38 |  |
|  |  | 6MWD (Follow-up) |  | 527.0(78.0), 30 |  | 497.0(76.0), 29 |  | 0.69 |  |
| Miozzo et al. (2018), In-patient | Double exercise therapy vs. single exercise therapy | 6MWD  (discharge) | High-intensity inspiratory muscle training and aerobic exercise (double exercise therapy) | 638.6(84.0), 9 | Aerobic exercise (single exercise therapy) | 620.0(85.6), 9 | 75% | 0.38 | None |
|  |  | Peak VO_2_ (discharge) |  | 20.9(4.2), 9 |  | 24.3(5.79), 9 |  | -1.16 |  |
| Santos et al. (2018), In-patient | High-intensity exercise (triple) therapy vs. low-intensity exercise (triple) therapy | 6MWD  (discharge) | Moderate to High-intensity inspiratory muscle training, Aerobic exercise, and resistance exercise (triple exercise therapy) | 537.9(56.8), 12 | Inspiratory muscle training, aerobic exercise, Upper and lower limb resistance exercise  (triple exercise therapy) | 459.1(53.0), 12 | 100% | 2.52 | Not available |
|  |  | Peak VO2 (discharge) |  | 23.6(1.1), 12 |  | 22.3(0.4), 12 |  | 2.76 |  |

Supplementary Table 4. Exercise protocol for CABG patients among the included article.

| Study | Control group (CG) | Intervention Group(s) |
| --- | --- | --- |
| Han et al. (2022) | Health education, a guideline for physical activity | SGR: CG + walking, abdominal and deep breathing, physiotherapy care.  IGR: CG + Walking, Deep breathing, physiotherapy care, Intensive care unit rehabilitation (head movement, sitting on bed, chair; sitting to standing, walking around the bed) |
| Eibel et al. (2022) | Vibrocompression, passive manual expiratory therapy, fractional inspiration in times, diaphragmatic breathing, active movement, walking, stair climbing. | Group 1: CG + Isometric Handgrip Resistance Exercise  Group 2: CG + ventilatory muscle training |
| Girgin et al. (2021) | Deep breathing, Coughing, Tapotement, Incentive spirometer, walking | Active Cycle of Breathing Techniques, Postural drainage, Lower and upper limb exercises, walking. |
| Zanini et al. (2019) | Deep breathing, Coughing or huffing, Active movement ankle and wrist, expiratory positive airway pressure | Group 1: CG + inspiratory muscle training, Active exercise for upper and lower limbs, Walking, Stepping up and down.  Group 2: CG + Active exercise for upper and lower limbs, Walking, Stepping up and down.  Group 3: CG + inspiratory muscle training |
| Mendes et al. (2009) | Breathing exercise, coughing | CG + Active and Active-assistive exercises of the lower and upper limbs, walking. |
| Busch et al. (2012) | Walking, calisthenics, cycling | CG + leg extension, leg press, leg curls using weight machines, and biceps curls using free weights, balance training. |
| Hirschhorn et al. (2008) | Health education, huffing/coughing, Postural change | Group 1: CG + walking  Group 2: CG + Walking, breathing exercise. |
| Miozzo et al. (2018) | Treadmill walking | CG + high-intensity inspiratory muscle training |
| Santos et al. (2018) | Inspiratory muscle training with constant low-intensity, treadmill walking, upper and lower limbs resistance training with dumbbells, shin guards and elastic bands. | Moderate-to-high intensity inspiratory muscle training, treadmill walking, upper and lower limbs resistance training with dumbbells, shin guards and elastic bands. |

Supplementary Table 5. GRADE quality assessment for Peak VO_2_.

| **Outcomes** | | **Total number of participants (No. of study)** | **GRADE**  **Certainty of the evidence ^a^** | **Anticipated absolute effects (95% CI) ^b^** | |
| --- | --- | --- | --- | --- | --- |
|  |  |  |  | **Mean value in Control group** | **Std. Mean difference in**  **Intervention group with control group** |
| Exercise capacity on Peak VO_2_ | | | | | |
| Multiple exercises. Vs. single exercise | | | | | |
| In-patient | Discharge | 126 (1) | ⊕⊕ΟΟ  LOW ^c^ | 13.6 mL/kg/min | -0.03 mL/kg/min (-0.38 to 0.32 mL/kg/min) |
|  | Follow-up | 39 (1) | ⊕⊕⊕Ο  MODERATE ^c, d^ | 17.3 mL/kg/min | 0.86 mL/kg/min (0.08 to 1.64 mL/kg/min) |
| Out-patient (discharge) | Double exercises Vs. single exercise | 18 (1) | ⊕⊕ΟΟ  LOW ^e, f^ | 24.3 mL/kg/min | -0.64 mL/kg/min (-1.59 to 0.31 mL/kg/min) |
|  | HI triple exercises Vs. LI triple exercise | 24 (1) | ⊕⊕⊕⊕  HIGH ^g^ | 22.3 mL/kg/min | 1.52 mL/kg/min (0.59 to 2.44 mL/kg/min) |

^a^ GRADE (**G**rading of **R**ecommendations, **A**ssessment, **D**evelopment, and **E**valuations) Working Group for grading of trial evidence.

High certainty refers to a high confident result where the true value is relatively close to that of the estimate of the effect. Moderate certainty refers to moderately confident in the effect estimate, the true value is seeming to be close to the estimated effect with a probable difference. Low certainty refers to less confidence due to limited effect and substantial difference in the estimated effect. Very low certainty refers to very less confidence in effect because the true effect seems to be substantially different from the estimated effect.

^b^ the risk in the intervention group and its 95% confidence interval [CI] and fixed effect was used to measure risk in the comparison group;

^c^ Problem on randomization.

^d^ Considerable heterogeneity found.

^e^ small sample size.

^f^ Concern with missing outcome data and reporting bias.

^g^ Did not find any problem with the risk of bias and the sample size was considerable.

mL=milliliter; kg= Kilogram; min= minute; MD= Mean difference; HI= High-intensity; LI= Low-intensity

Supplementary Table 6. PEDro scores for included studies.

| **Study** | **Criterion 02: Random allocation** | **Criterion 03: Concealed allocation** | **Criterion 04:**  **Groups similar at baseline** | **Criterion 05: Participant  blinding** | **Criterion 06: Therapist  blinding** | **Criterion 07: Assessor blinding** | **Criterion 08:**  **more than 85% of outcomes measured** | **Criterion 09: Intention-to-treat analysis** | **Criterion 10: Between-group  difference  reported** | **Criterion 11:**  **Point estimate  and variability  reported** | **Total (0 to 10)** |
| --- | --- | --- | --- | --- | --- | --- | --- | --- | --- | --- | --- |
| Han et al. (2022) | Y | Y | Y | Y | Y | N | Y | Y | Y | Y | 9 |
| Eibel et al. (2022) | Y | Y | Y | Y | N | N | Y | Y | Y | Y | 8 |
| Girgin et al. (2021) | Y | N | Y | N | N | N | Y | Y | Y | Y | 6 |
| Zanini et al. (2019) | Y | Y | Y | Y | N | Y | Y | Y | Y | Y | 9 |
| Mendes et al. (2009) | Y | Y | Y | Y | N | Y | Y | Y | Y | Y | 9 |
| Busch et al. (2012) | Y | Y | Y | Y | Y | Y | Y | Y | Y | Y | 10 |
| Hirschhorn et al. (2008) | Y | N | N | N | N | Y | Y | Y | Y | Y | 6 |
| Miozzo et al. (2018) | Y | Y | N | Y | N | N | Y | Y | Y | Y | 7 |
| Santos et al. (2018) | Y | Y | Y | Y | N | N | Y | Y | Y | Y | 8 |
